# Case Report: Immunophenotypically diverse immature patterns, including variable TdT expression, in aggressive B-cell lymphomas and leukemia with *MYC* rearrangement

**DOI:** 10.3389/fonc.2025.1684005

**Published:** 2025-10-09

**Authors:** Daishi Kato, Takahiro Fujino, Reiko Isa, Haruya Okamoto, Taku Tsukamoto, Shinsuke Mizutani, Yuji Shimura, Tsutomu Kobayashi, Hitoji Uchiyama, Masafumi Taniwaki, Aya Miyagawa-Hayashino, Junya Kuroda

**Affiliations:** ^1^ Division of Hematology and Oncology, Department of Medicine, Kyoto Prefectural University of Medicine, Kyoto, Japan; ^2^ Department of Hematology, Japanese Red Cross Kyoto Daiichi Hospital, Kyoto, Japan; ^3^ Department of Pathology and Applied Neurobiology, Kyoto Prefectural University of Medicine, Kyoto, Japan

**Keywords:** B-cell lymphoma, MYC rearrangement, terminal deoxynucleotidyl transferase, CD20, immunophenotypic immaturity, surface membrane immunoglobulin

## Abstract

This study is a retrospective analysis of our case series and literature review of aggressive B-cell lymphomas with *MYC* rearrangements that show immunophenotypic immaturity, including expression of terminal deoxynucleotidyl transferase (TdT), weak or negative CD20, and the absence of surface membrane immunoglobulin (smIg) and light chains. Although classified as diffuse large B-cell lymphoma (DLBCL) or high-grade B-cell lymphoma (HGBCL), these cases sometimes resemble B-lymphoblastic leukemia/lymphoma (B-ALL/LBL) immunophenotypically, creating diagnostic ambiguity. We report four cases: three diagnosed as HGBCL-*MYC/BCL2* with TdT expression, and one as B-ALL with *MYC* rearrangement. Case 1 developed from follicular lymphoma with TdT-positive blastoid transformation. Case 2 showed widespread disease, complex cytogenetics, weak TdT positivity, and absence of light chain. Case 3 displayed scattered TdT expression and surface light chain restriction. Case 4, initially diagnosed as a mature B-cell neoplasm, was ultimately reclassified as B-ALL with *MYC* rearrangement and focal TdT expression. In all cases, CD20 expression was weak or negative. These overlapping immunophenotypes between mature B-cell neoplasms and lymphoblastic leukemia were also documented in previous reports of 89 patients with various *MYC*-rearranged mature B-cell lymphoma subtypes. Through review, we identified the patient population with tumor cells showing less than 10% positive TdT expression, weak or negative CD20, and absence of smIg and light chain expression. Conversely, B-ALL with *MYC* rearrangement also exhibits aberrant immunophenotypes, such as negative to weak TdT expression and variable CD20, smIg, or light chain expression. Notably, this phenotypic immaturity appears closely linked to the presence of *MYC* rearrangement. In contrast, recent studies have shown that TdT-positive DLBCL/HGBCL-*MYC/BCL2* and B-ALL with *MYC* rearrangement have distinct molecular features. In conclusion, an analysis of 93 cases, including our four cases, suggests that *MYC* rearrangement contributes to immature immunophenotypic profiles in both lymphoma and leukemia, emphasizing the importance of a refined classification that integrates morphology, immunophenotype, and genetics.

## 1 Introduction

About 2% of cases of diffuse large B-cell lymphoma (DLBCL) or high-grade B-cell lymphoma (HGBCL) with *MYC* and *BCL2* rearrangements show expression of terminal deoxynucleotidyl transferase (TdT) (1). These cases also exhibit other immature immunohistochemical features, such as the weak/absence of CD20 expression, lack of surface membrane immunoglobulin (smIg), and no light chain detection. There is ongoing debate over whether these lymphomas should be classified as B-lymphoblastic leukemia/lymphoma (B-ALL/LBL) or DLBCL/HGBCL (2–4). However, the 5th WHO classification now categorizes them as DLBCL/HGBCL-*MYC/BCL2* (with or without *BCL6* rearrangement) with TdT expression (5). Conversely, differential diagnosis should consider a rare B-ALL/LBL variant with *MYC* rearrangement, displaying characteristics of mature B-cell lymphomas (BCLs), such as CD20 positivity, TdT negativity, smIg expression, light chain restriction, and the *IGH-BCL2* translocation (5). In *MYC*-rearranged B-cell precursor acute lymphoblastic leukemia (BCP-ALL), which is common in pediatric cases and typically characterized by the absence of smIg and light chains, some instances have also shown characteristics of mature B cells (6).

We present a case of HGBCL-*MYC/BCL2* with TdT expression that evolved from FL, along with two other cases of HGBCL-*MYC/BCL2* with TdT and one case of B-ALL with *MYC* rearrangement, all diagnosed and treated at our institutions. Additionally, we reviewed previously reported cases of aggressive BCLs (1–4, 7–15), B-ALL/LBL (16–24), and BCP-ALL (6, 25–33) with *MYC* rearrangement that exhibit immunophenotypically diverse immature patterns, including varying TdT, CD20, smIg, and light chain expressions.

## 2 Case report

### 2.1 Case 1

**Figure 1:**
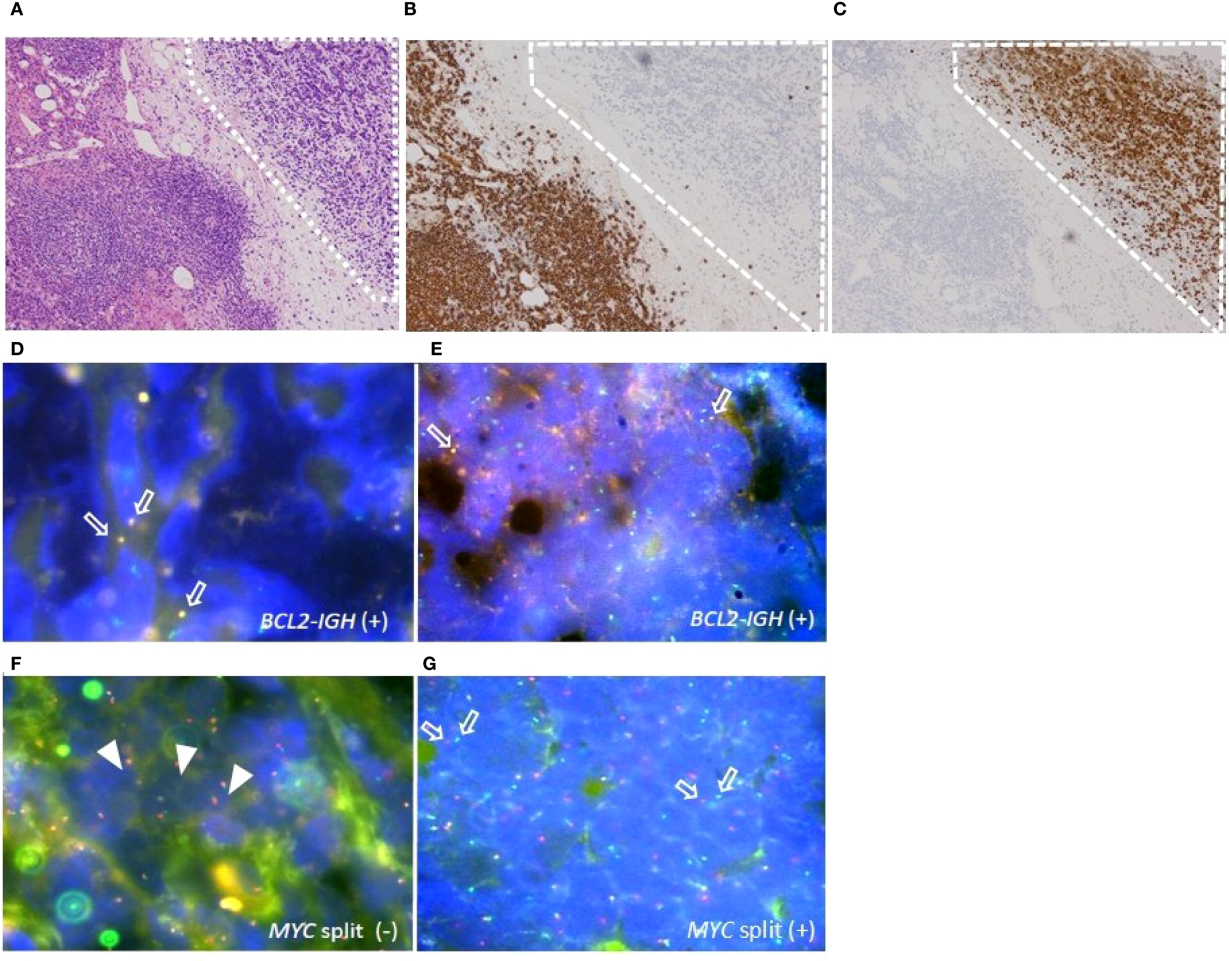
Histopathologic features of the adipose tissue around the biopsied lymph nodes in case 1. **(A)** B-lymphoblastic lymphoma (LBL) component (surrounded by dashed square) examined by Hematoxylin-Eosin (HE) staining, original magnification ×40. **(B, C)** IHC staining for CD20 **(B)** and TdT **(C)** in B-LBL component (surrounded by dashed square), original magnification ×40. **(D–G)** Tissue fluorescence *in situ* hybridization (t-FISH) analyses for *IGH/BCL2* and *MYC* rearrangement. **(D, E)** t-FISH for *IGH/BCL2* in FL component **(D)** and in B-LBL component **(E)**. Arrows indicate the fusion signals of *IGH/BCL2*. **(F, G)** t-FISH for *MYC* split analyses in FL component **(F)** and in B-LBL component **(G)**. Arrowheads indicate the fusion, and arrows indicate the split signals of *c-MYC* gene.

A 76-year-old woman visited our hospital with low back pain linked to intraperitoneal lymphadenopathy exhibiting 18F-fluoro-2-deoxy-D-glucose (18F-FDG) uptake on positron emission tomography–computed tomography (PET/CT). The biopsy of the affected lymph nodes showed two distinct histological components. The central region of the lymph nodes was marked by multiple follicles containing proliferating small to medium-sized atypical lymphocytes. Notably, each follicle housed 6–15 centroblasts viewed under high-power microscopy, and a follicular dendritic cell meshwork surrounded the nodules (**Supplementary Figure S1A**). In addition, a group of atypical lymphoid cells with condensed chromatin was also present in the adipose tissue around the lymph nodes (**Figure 1A**). In the initial lesion, atypical lymphoid cells tested positive for CD10, CD20, CD21, and BCL-2 (**Supplementary Figure S1B**) but were negative for CD3, confirming the diagnosis of FL grade 2. In the subsequent lesion, atypical lymphoid cells tested negative for CD3 and CD20 (lesion surrounded by dashed square in **Figure 1B**), while being positive for CD10, CD79a, BCL-2, PAX-5, Ki-67, and TdT (**Figure 1C**), indicating the presence of a B-LBL component. Tissue fluorescence *in situ* hybridization (t-FISH) using the Vysis LSI *IGH/BCL2* Dual Color Fusion Probe (Abbott, Abbott Park, IL) showed fusion signals for *IGH* and *BCL2* in both the FL and B-LBL components (**Figures 1D, E**). Additionally, *MYC* rearrangement was observed only in the B-LBL component, detected by t-FISH with the Vysis LSI *MYC* Dual Color Break Apart Rearrangement Probe (Abbott) (**Figures 1F, G**) (**Table 1**). The bone marrow (BM) examination revealed the presence of a small number of CD20-positive but TdT-negative atypical lymphoid cells, suggesting infiltration of the BM by the FL component. According to the Ann Arbor disease staging system, we diagnosed Case 1 as stage IV HGBCL-*MYC/BCL2* with TdT expression, transformed from FL. Although the patient temporarily responded to G-CHOP therapy, comprising obinutuzumab, cyclophosphamide, doxorubicin, vincristine, and prednisolone, the disease relapsed systemically after five cycles, without FL components. Presently, the patient is receiving salvage chemotherapy with fractionated cyclophosphamide, vincristine, doxorubicin, and dexamethasone (hyper-CVAD) alternating with high-dose methotrexate and cytarabine therapy, as blinatumomab was ineffective.

**Table 1:** Clinical, cytogenetic, and immunophenotypic manifestation of aggressive B-cell lymphomas and B-ALL with concomitant *MYC* rearrangement in our hospitals.

Case	Age /Gender	Disease subtype	Ann Arbor disease stage (lymphoma lesion)	Chromosomal rearrangement			Immunohistochemistry	Flow cytometry
				*MYC*	*BCL2*	other		
1	76/F	tFL	IVA (BM, mesenteric LN)	+	+	–	LN: CD20-, CD5-, CD3-, CD10+, CD79a+, TdT+ (100%), PAX5+, BCL-2+, BCL-6+ (50%) BM: CD20+ (scatter), TdT+ (scatter)	LN: CD5-, CD10+, CD19+, CD20+ (62.5%), CD22-, λ+ BM: CD5-, CD10+, CD19+, CD20+ (69.3%), CD22+, CD34-, CD79a+, TdT+ (21.9%), λ+
2	80/F	HGBCL	IVB (BM, retroperitoneal LN, heart, stomach, adrenal gland, kidney, bone)	+	+	*BCL6* rearrangement	LN: CD3-, CD5-, CD20+ (10%), CD79a+, CD10+, BCL-2+, BCL-6+ (90%), TdT+ (5%) BM: data not available	LN: CD5-, CD10+, CD19+, CD20 (0.9%), CD22-, CD79a+, TdT- (0.9%), lc- BM: data not available
3	73/M	HGBCL	IVB (BM, retroperitoneal LN, para-aortic LN, hepatic hilar LN、heart)	+	+	–	LN: CD3-, CD5-, CD10+, CD20 -, CD34-, CD38+, CD79a+, CD138-, BCL-2+, BCL-6– (10%), EBER-, TdT+ (~5%), PAX5+ BM: CD3-, CD10+, CD38+, CD79a+, TdT-	LN: CD5-, CD10+, CD19+, CD20 (4.5%), CD22+, CD34-, CD38+, CD79a+, λ+ BM: CD1a– (0.5%), CD5-, CD10+, CD19+, CD20– (9.6%), CD22+, CD34–, CD38+, CD138-, TdT– (1.2%)、lc-
4	85/M	B-ALL	IVB (BM)	+	–	–	CD10+, CD20– (5%), CD38+, CD79a+, TdT+ (50%), BCL-2-	CD1a–, CD5-, CD10+, CD19+, CD20–(1.5%), CD38+, CD22+, CD79a+, CD34–, TdT– (1.1%), λ+

tFL, transformed follicular lymphoma; HGBCL, high-grade B-cell lymphoma; B-ALL, B-cell acute lymphoblastic leukemia; M, male; F, female; BM, bone marrow; LN, lymph node; lc, light chain.

### 2.2 Case 2

**Figure 2:**
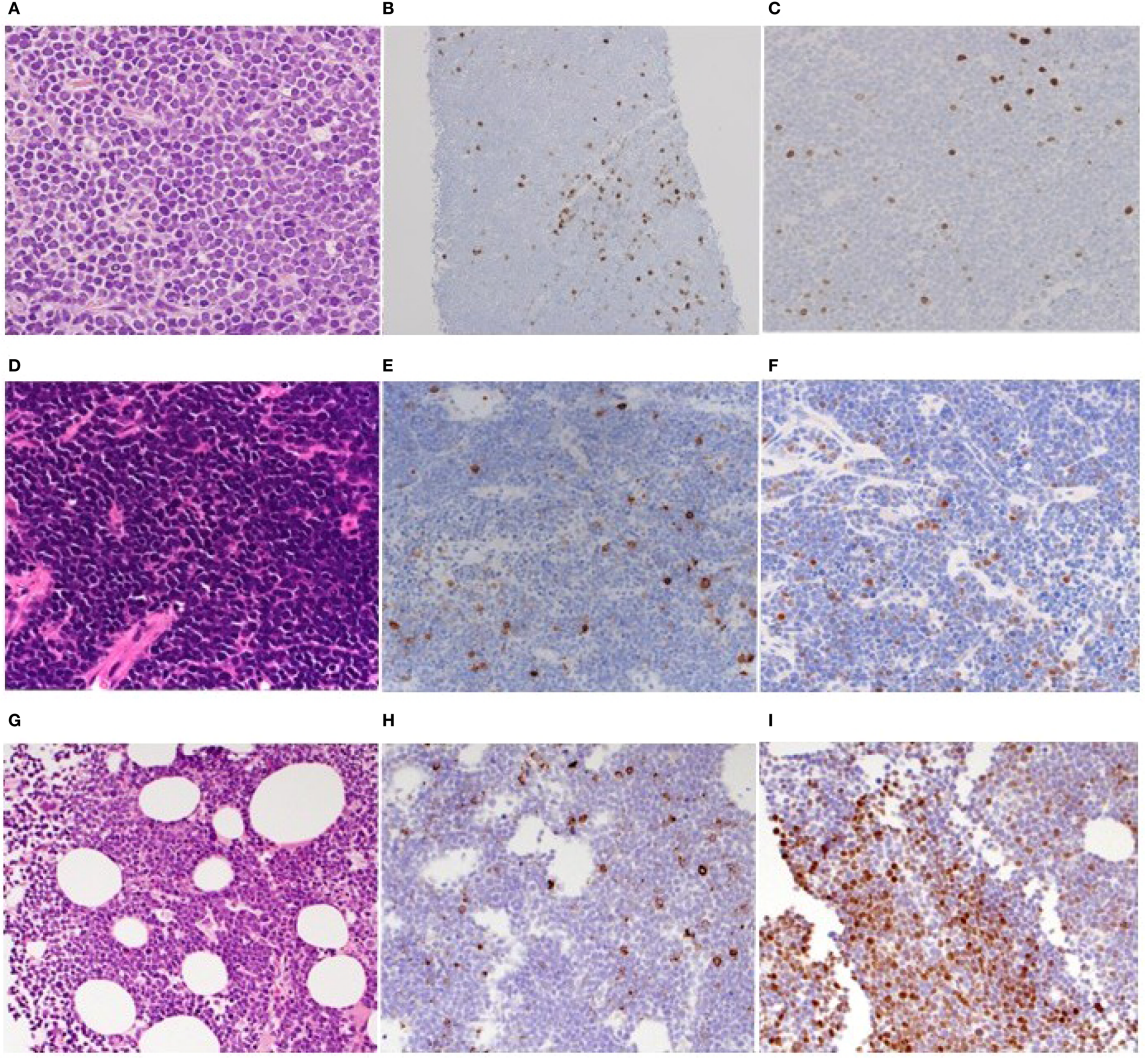
Histopathologic features of the biopsied specimens of cases 2-4. **(A–C)**. Retroperitoneal tumorous lesion of case 2. HE-stained biopsied specimen (×100) **(A)** and IHC staining for CD20 **(B)** and TdT (×40) **(C)**. **(D–F)**. Retroperitoneal tumorous lesion of case 3. HE-stained biopsied specimen (×100) **(D)** and IHC staining for CD20 **(E)** and TdT (×40) **(F)**. **(G–I)**. Bone marrow of case 4. HE-stained biopsied specimen (×100) **(G)** and IHC staining for CD20 **(H)** and TdT (×40) **(I)**.

An 80-year-old woman was referred to our hospital due to symptoms of systemic decline, including chest discomfort, loss of appetite, swelling in her lower legs, and back pain lasting several weeks. PET/CT scans revealed widespread abnormal 18F-FDG-avid lesions across multiple sites: bilateral adrenal glands, pancreas, perinephric and right femoral lumbar muscles, pelvic cavity, atrial septum, soft tissue around the Th3 vertebral body, uterine endometrium, and bones. A biopsy of the retroperitoneal tumor showed proliferation of small, atypical lymphoid cells with high nuclear-to-cytoplasm ratio (**Figure 2A**; **Supplementary Figure S2A**), positive for CD10, CD19, CD79a, BCL-2 (**Supplementary Figure S2B**), BCL-6 (**Supplementary Figure S2C**), and c-MYC (**Supplementary Figure S2D**) by immunohistochemistry. About 10% of tumor cells tested positive for CD20 (**Figure 2B**), and 5% were weakly positive for TdT (**Figure 2C**). Flow cytometry analysis showed that the tumor cells did not express smIg. Chromosomal analysis with G-banding indicated a complex abnormality: 50, XX, add(5)(q11.2),?t(8;14)(q24;q32), +12, t(14;18)(q32;q21), +20. The standard double-color FISH tests confirmed that all tumor cells had translocations involving *IGH/BCL2* and *IGH/MYC*, along with a *BCL6* gene rearrangement (**Table 1**). Overall, we diagnosed Case 2 with stage IV HGBCL-*MYC/BCL2* and *BCL6* rearrangements, along with an unusual TdT-positive component. Despite treatment with EPOCH chemotherapy, including doxorubicin, vincristine, etoposide, cyclophosphamide, and prednisolone, the disease progressed, and the patient died on day 41.

### 2.3 Case 3

A 73-year-old man presented to our hospital with two months of fatigue and one week of left lower abdominal pain. Blood tests indicated elevated LDH levels and kidney impairment. An enhanced-contrast CT scan showed a large mass in the left retroperitoneal area involving the left ureter, along with enlarged lymph nodes around the right hepatic artery and near the aorta. Biopsy of the retroperitoneal mass revealed proliferation of small to medium atypical lymphoid cells with high nuclear-to-cytoplasm ratios (**Figure 2D**; **Supplementary Figure S3A**). These cells tested positive for CD10, CD79a, PAX-5, BCL-2, BCL-6 (10-20%), and c-MYC (**Supplementary Figures S3B–G**), but most tumor cells were negative for CD20 (**Figure 2E**) on immunohistochemistry. Of note, about 5% of the tumor cells scatteredly tested positive for TdT (**Figure 2F**). Flow cytometry analysis showed that the tumor cells did not express smIg. In addition, Epstein-Barr virus early RNA *in situ* hybridization (data not shown). Chromosomal analysis via G-banding identified a complex abnormality including a 48, XY karyotype with multiple deletions, additions, and translocations, including -2, -6, -8, -13, -14, t(14;18)(q32;q21), -17, -19, +21, and other marker chromosomes. Conventional double-color FISH confirmed that all tumor cells carried gene rearrangements of *BCL2* and *MYC* (**Table 1**). Based on our findings, we diagnosed the case asstage IV HGBCL-*MYC/BCL2* with abnormal TdT expression. Despite the patient receiving EPOCH chemotherapy, the disease returned within three months. We then gave the patient DeVIC therapy, which included dexamethasone, etoposide, ifosfamide, and carboplatin, as a temporary treatment; however, new lesions developed in the lung and hilar lymph nodes. The patient was then treated with CAR-T therapy, and they have since stayed in remission with a complete response.

### 2.4 Case 4

An 85-year-old man was referred to our hospital, presenting with fever and fatigue. Laboratory tests showed elevated LDH levels and increased white blood cell counts. Peripheral blood and bone marrow samples contained approximately 20% and 80% abnormal small lymphocytes, respectively. There was no lymphadenopathy or splenomegaly on plain CT. Flow cytometry of bone marrow cells identified a CD45-dim population positive for CD10, CD19, CD79a, CD22, CD38, and light chain restriction for kappa, while negative for CD1a, CD3, CD34, TdT, and CD20. Due to rapid progression, we initially diagnosed it as a stage IV CD20-negative mature B-cell neoplasm and treated it with prednisolone. Unfortunately, he died from alveolar hemorrhage caused by disseminated intravascular coagulation on hospital day 3. Subsequently, biopsy revealed the bone marrow was filled with blastoid cells (**Figure 2G**) immunohistochemically positive for CD20 (partial) (**Figure 2H**), CD10, CD79a (**Supplementary Figures S4A, B**), CD38, TdT (focal, about 50%) (**Figure 2I**), and c-MYC (**Supplementary Figure S4C**), but negative for BCL-2 (**Supplementary Figure S4D**). FISH analysis showed split signals for *MYC* and no *IGH-BCL2* fusion. G-banding revealed complex chromosomal abnormalities, including additions at 1(p11), 3(p21), deletions on chromosomes 15 and 19, and other unspecified alterations (**Table 1**). The final diagnosis was B-ALL with *MYC* rearrangement.

## 3 Discussion

Although the first three cases in our series were initially identified as mature B-cell malignancies, their tumor cells displayed immunophenotypic features typical of immature B cells. These cells were weakly or negatively stained for CD20, lacked smIg and light chains, and showed variable TdT expression, including scattered, weak, or diffuse patterns. Our four cases of BCL showed cytogenetic abnormalities involving either *MYC* and *BCL2* rearrangements or combinations of *MYC*, *BCL2*, and *BCL6* rearrangements. This contrasts with earlier studies that reported multiple cases of BCL with an immature phenotype and only a *MYC* rearrangement (1, 2, 4, 7, 9, 16). Furthermore, many BCL cases with *MYC* and *BCL2* rearrangements are derived from transformed follicular lymphoma (FL), where the *MYC* rearrangement typically occurs during transformation. Our findings overall indicate that *MYC* rearrangement is more likely to result in abnormal immature cell types than other genetic defects. We gathered case reports and series of aggressive B-cell lymphomas (including DLBCL, HGBCL, Burkitt lymphoma, mantle cell lymphoma, and B-lymphoblastic lymphoma) from PubMed that showed *MYC* rearrangement through FISH or G-banding, and displayed unusual immature B-cell features like TdT positivity on immunohistochemistry, CD20 negativity, or loss of flow cytometric smIg. (**Supplementary Table S1**). In this context, TdT expression in BCLs varies from scattered and weak to diffuse, unlike the diffuse pattern typical of B-ALL. In 49 reported cases, including our three patients, TdT-positive BCL cells often lack smIg, show light chain restriction, and are CD20 positive. These features can be misleading but usually indicate the presence of both “immature” tumor components and mature B-cell components. Several studies have highlighted this as a diagnostic challenge (1, 3, 4, 8). Typically, TdT positivity is considered when 10% or more of tumor cells are positive in immunohistochemistry (34), but in mature BCLs with *MYC* rearrangement, this should be interpreted with caution. Previous studies on double/triple hit lymphoma and HGBCL with *MYC* rearrangement have reported cases with a few percent of TdT-positive cells, rather than being negative, due to the presence of immature B-cell features despite TdT expression below 10%. (3, 7, 8, 35, 36). On the other hand, cases of HGBCL/DLBCL-*MYC/BCL2* and HGBCL with *MYC* rearrangement may show no TdT expression, along with missing smIg, light chain, and CD20. This highlights the variable TdT expression in immature cells found within aggressive mature B-cell lymphomas (3, 9, 37–39).

These irregular marker patterns, similar to those in ALL, are evident in our case 4, where neoplastic lymphoid cells displayed weak CD20 and focal TdT expression by immunohistochemistry, and flow cytometry showed light chain expression in tumor cells. This underscores the complexity of immunophenotypic profiles.

These phenotypes were also observed in BCP-ALL cases with *MYC* rearrangement (6, 25–33). We chose B-ALL/BCP-ALL cases that had previously been reported, where *MYC* rearrangement was confirmed by FISH or G-banding, and TdT expression was evaluated by flow cytometry (**Supplementary Table S2**). Several studies show that *MYC* rearrangement affects abnormal expression patterns of TdT, CD20, smIg, and light chains in B-ALL (6, 17–33). Looking back at the UKALLXII/ECOG-ACRIN E2993 study, researchers found that B-ALL cases with *MYC* rearrangement show significant diversity, including cases that are both TdT-positive and -negative, as well as smIg-positive and -negative (40). Reports have also documented TdT-positive and smIg-positive B-ALL cases with *MYC* rearrangements (17–24). Additionally, a study on pediatric BCP-ALL found that cases without smIg and light chain expression, and with *MYC* rearrangement, had similar variability in CD20 expression across Japanese and international cohorts. However, TdT expression was less common in the Japanese group and more prevalent internationally (6). Additionally, weak TdT expression has been observed in several cases of *MYC*-rearranged B-ALL and BCP-ALL (19, 24, 25, 30). According to other genetic studies, BCLs with *MYC* rearrangement often contain genetic abnormalities linked to mature B-cell lymphomas (1), whereas B-ALL with *MYC* rearrangement often lacks additional mutations (41). Since both types show abnormal immature B-cell features despite different genetic characteristics besides *MYC* rearrangement, it is suggested that the *MYC* rearrangement may play a role in this phenomenon.

## 4 Limitation

Our analysis has several limitations. This study is a case series that brings together previously reported cases to get a deeper understanding of the disease. However, for statistical analysis, problems like unclear disease definition, a small sample size, and potential biases still exist, and these need to be addressed in future studies. It’s based on studies of the immune system and cells after the fact, but the specific molecular genetic mechanisms behind B-cell maturation with *MYC* rearrangement are still unknown. Additionally, evaluating TdT staining can be somewhat subjective. For example, in previous B-ALL studies, some cases without TdT might still have fewer than 10% TdT-positive cells, while others might be considered TdT-positive even if less than 10% of tumor cells express TdT. As shown in the table, B-ALL/BCP-ALL phenotypes are mainly evaluated using flow cytometry, whereas BCLs are primarily evaluated using immunohistochemistry. This difference in evaluation methods should also be carefully noted. Additionally, in BCLs, cases that show weak TdT positivity in immunohistochemistry may appear negative in flow cytometry, showing how the two methods can produce different results. In pediatric B-ALL with *MYC* rearrangement, cases with smIg expression are rare. The specific case also involved an *MLL* rearrangement (23). Since B-ALL cases with *MLL* rearrangements and smIg positivity have been reported separately (42), we cannot exclude the possibility that *MLL* rearrangements also contribute to B-cell immaturity. Regarding treatment, CAR-T therapy was successful in Case 3. Multiple studies have demonstrated the effectiveness of CAR-T therapy for relapsed or refractory HGBCL with DHL/THL (43, 44). However, only one case with TdT expression has been reported (45), and in that instance, the patient relapsed after CAR-T therapy, which differs from Case 3. We’re looking forward to more such cases being reported.

## 5 Conclusion

We propose that *MYC* rearrangement might play a role in the varying levels of immaturity seen in B-cell lymphoma/leukemia, irrespective of cell origin or other gene mutations. This is evidenced by findings such as weak or missing CD20, negative smIg and light chain, and TdT expression that varies from absent, scattered, weak, focal, to diffuse.Even under the current WHO classification diagnostic criteria, there are some cases of *MYC*-rearranged B-cell lymphoma/leukemia with aberrant immature features in which it is difficult to determine whether the disease should be diagnosed as B-ALL/LBL or mature BCL. One possible fundamental reason for this diagnostic challenge is that *MYC* rearrangement may drive B cells to exhibit both tumor proliferative capacity and aberrant immaturity.

## Data Availability

The datasets presented in this article are not readily available because data supporting this study’s findings are available from the corresponding author upon reasonable request. However, due to privacy or ethical restrictions, the data are not publicly available. Requests to access the datasets should be directed to Junya Kuroda, junkuro@koto.kpu-m.ac.jp.

## Supplementary material

Supplementary Figure 1 Histopathologic features of the central region of biopsied lymph node in case 1. **(A)**. Follicular lymphoma (FL) component examined by Hematoxylin-Eosin (HE) staining, original magnification ×40. **(B)**. FL component examined by the immunohistochemical staining (IHC) for BCL-2, original magnification ×40.

Supplementary Figure 2 Histopathologic features of the biopsied retroperitoneal tumorous lesion in case 2. **(A)**. HE-stained biopsied specimen, original magnification ×40. **(B–D)**. IHC examinations for BCL-2 (×40) **(B)**, BCL-6 (×40) **(C)**, and c-MYC (×40) **(D)**.

Supplementary Figure 3 Histopathologic features of the biopsied retroperitoneal tumorous lesion in case 3. HE-stained biopsied specimen, original magnification (×40) **(A)**, and IHC examinations for CD10 (×40) **(B)**, CD79a (×40) **(C)**, PAX-5 (×40) **(D)**, BCL-2 (×40) **(E)**, BCL-6 (×40) **(F)**, and c-MYC (×40) **(G)**.

Supplementary Figure 4 Histopathologic features of the bone marrow biopsied specimen in case 4. IHC examinations for CD10 (×40) **(A)**, CD79a (×40) **(B)**, MYC (×40)**(C)**, and BCL-2 (×40) **(D)**.

Supplementary Table 1 Clinical, cytogenetic, and immunophenotypic manifestation of aggressive B-cell lymphomas with concomitant *MYC* rearrangement.

Supplementary Table 2 Clinical, cytogenetic, and immunophenotypic manifestation of B-ALL and BCP-ALL with concomitant *MYC* rearrangement.
