# Supplementary figures and images for: Case Report: Immunophenotypically diverse immature patterns, including variable TdT expression, in aggressive B-cell lymphomas and leukemia with MYC rearrangement
Source: Front Oncol. 2025 Oct 9;15:1684005. doi: 10.3389/fonc.2025.1684005 (PMC12545147; doi:10.3389/fonc.2025.1684005)

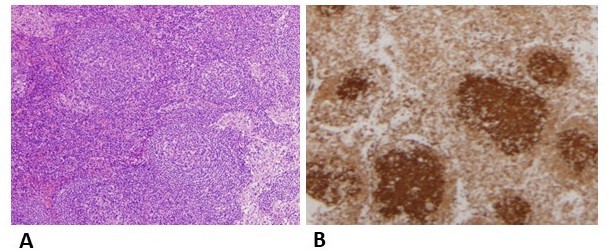

Supplement: Supplementary Figure 1 — Histopathologic features of the central region of biopsied lymph node in case 1. (A). Follicular lymphoma (FL) component examined by Hematoxylin-Eosin (HE) staining, original magnification ×40. (B). FL component examined by the immunohistochemical staining (IHC) for BCL-2, original magnification ×40. [file Image1.jpeg]

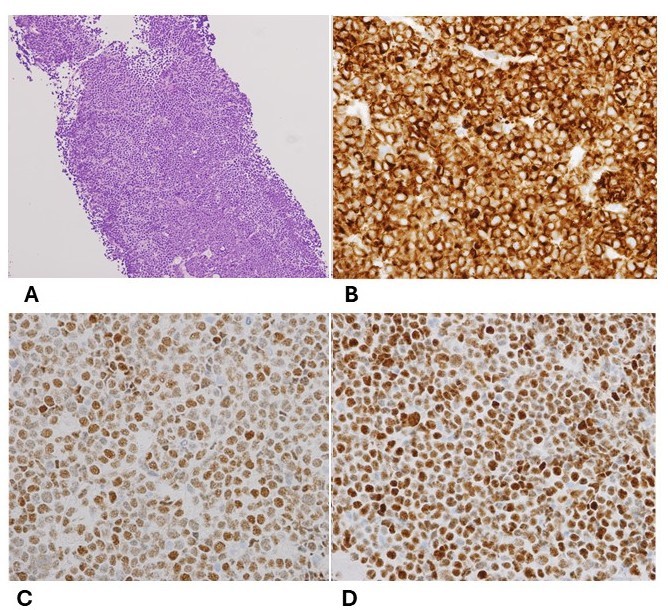

Supplement: Supplementary Figure 2 — Histopathologic features of the biopsied retroperitoneal tumorous lesion in case 2. (A). HE-stained biopsied specimen, original magnification ×40. (B–D). IHC examinations for BCL-2 (×40) (B), BCL-6 (×40) (C), and c-MYC (×40) (D). [file Image2.jpeg]

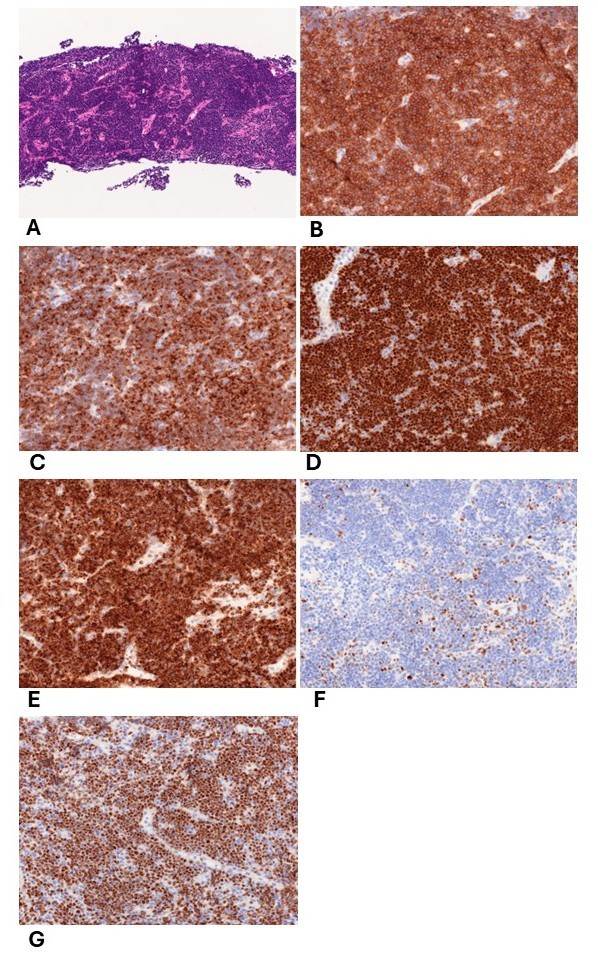

Supplement: Supplementary Figure 3 — Histopathologic features of the biopsied retroperitoneal tumorous lesion in case 3. HE-stained biopsied specimen, original magnification (×40) (A), and IHC examinations for CD10 (×40) (B), CD79a (×40) (C), PAX-5 (×40) (D), BCL-2 (×40) (E), BCL-6 (×40) (F), and c-MYC (×40) (G). [file Image3.jpeg]

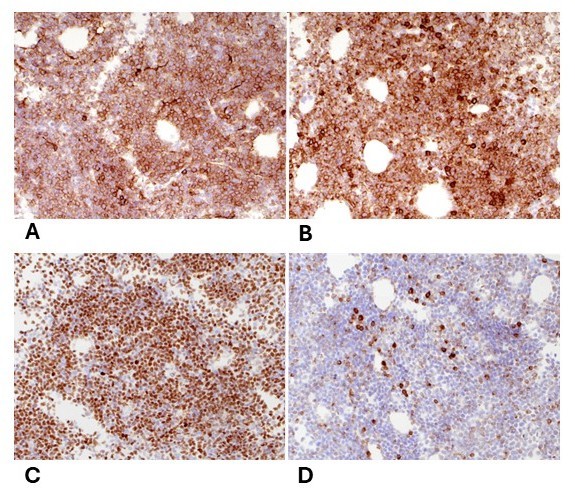

Supplement: Supplementary Figure 4 — Histopathologic features of the bone marrow biopsied specimen in case 4. IHC examinations for CD10 (×40) (A), CD79a (×40) (B), MYC (×40)(C), and BCL-2 (×40) (D). [file Image4.jpeg]
