# Supplementary material for: Case Report: Immunophenotypically diverse immature patterns, including variable TdT expression, in aggressive B-cell lymphomas and leukemia with MYC rearrangement
Source: Front Oncol. 2025 Oct 9;15:1684005. doi: 10.3389/fonc.2025.1684005 (PMC12545147; doi:10.3389/fonc.2025.1684005)
Supplement: Supplementary Table 1 — Clinical, cytogenetic, and immunophenotypic manifestation of aggressive B-cell lymphomas with concomitant MYC rearrangement. [file Table1.docx]

**Table S1. Clinical, cytogenetic, and immunophenotypic manifestation of aggressive B-cell lymphomas with concomitant *MYC* rearrangement**

| **No.** | **Age**  **/gender** | **Histopathological subtype** | **Chromosomal rearrangement** | | | **Immunophenotype** | | | **Ref.** |
| --- | --- | --- | --- | --- | --- | --- | --- | --- | --- |
|  |  |  | ***MYC*** | ***BCL2*** | **other** | **TdT**  **(IHC/FCM)** | **CD20**  **(IHC/FCM)** | **smIg** |  |
| 1 | 76/F | tFL | + | + | - | +100%/+ | -/+ | +λ | present cases  1-3 |
| 2 | 80/F | HGBCL | + | + | *BCL6*-r | +5%/- | +10%/- | - |  |
| 3 | 73/M | HGBCL | + | + | - | +~5%/- | -/- | + λ |  |
| 4 | 67/F | HGBCL | + | + | *BCL6*-r | +10~30%/NA | -/NA | NA | 1 |
| 5 | 62/F | tFL | + | + | - | +60-70%/NA | -/NA | + λ |  |
| 6 | 55/M | tFL | + | + | - | +>50%/NA | variable/NA | NA |  |
| 7 | 72/F | DHL | + | + | - | +20~30%/NA | variable/NA | +κ |  |
| 8 | 77/F | DHL | + | - | *BCL6*-r | +10~20%/NA | variable/NA | - |  |
| 9 | 62/F | HGBCL | + | - | - | +>10%/NA | +/NA | +κ |  |
| 10 | 50s/M | HGBCL | + | - | - | +/+dim | -/- | +κ | 2 |
| 11 | 74/F | tFL | + | + | - | +/+ | -/- | - | 3 |
| 12 | 59/M | BCLU | + | + | - | +/- | +/+ | + λ |  |
| 13 | 48/F | HGBCL | + | + | *BCL6*-r | +*/- | -/+ | +κ |  |
| 14 | 39/F | B-LBL | + | + | *BCL6*-r | +/+ | NA/- | +λ |  |
| 15 | 55/F | HGBCL | + | + | *BCL6*-r | +/+ | +/+ | +κ |  |
| 16 | 52/F | DLBCL | + | + | *BCL6*-r | -/NA | +/+dim | - |  |
| 17 | 57/M | DHL | + | + | - | +** | +** | +lc | 4 |
| 18 | 60/M | DHL | + | + | - | +** | +** | +lc |  |
| 19 | 55/M | THL | + | + | *BCL6*-r | +/NA | +/NA | +lc |  |
| 20 | 64/M | THL | + | + | *BCL6*-r | +/NA | +/NA | +lc |  |
| 21 | 43/M | DHL | + | + | - | +/NA | -/NA | NA |  |
| 22 | 61/F | THL | + | + | *BCL6*-r | +** | -** | NA |  |
| 23 | 50/M | tFL | + | + | - | NA/+ | NA/- | +lc |  |
| 24 | 78/F | tFL | + | + | - | +/+ | -/NA | +lc |  |
| 25 | 71/M | EBV+DLBCL | + | - | - | +/NA | -** | - |  |
| 26 | 33/F | tFL | + | + | - | <5%/NA | +/NA | +λ | 7 |
|  |  | tFL | + | + | - | +/NA | NA/NA | +λ |  |
| 27 | 62/F | tFL | + | + | - | +<1%/NA | NA/NA | +κ |  |
|  |  | tFL | + | - | - | +/NA | NA/NA | - |  |
| 28 | 67/M | tFL | + | - | - | +/NA | NA/NA | - |  |
| 29 | 69/M | tFL | + | + | - | +/NA | NA/NA | - |  |
| 30 | 68/M | tFL | + | + | - | +/NA | -/NA | +κ |  |
| 31 | 81/M | MCL | + | NA | *CCND1*-r | +20%/NA | NA/NA | NA | 8 |
| 32 | 71/M | MCL | + | NA | *CCND1*-r | +≦2%/NA | NA/NA | NA |  |
| 33 | 53/M | tMCL | + | NA | *CCND1*-r | +<5%/NA | NA/NA | NA |  |
| 34 | 54/M | EBV+BL | + | - | - | -/- | -/- | - | 9 |
| 35 | 39/M | tFL | + | + | - | -/- | +/+ | +λ | 10 |
|  |  | tFL | + | + | - | +/NA | -/NA | +λ |  |
|  |  | tFL | NA | NA | NA | +partially/NA | -/- | +λ |  |
| 36 | 66/M | EBV+tFL | + | + | *BCL6*-r | +** | -** | - | 11 |
| 37 | 86/F | EBV+tFL | + | - | *BCL6*-r | -** | +** | - |  |
| 38 | 56/M | BCLU | + | + | *BCL6*-r | - (5%)/NA | -** | +lc | 12 |
| 39 | 52/M | tFL | + | + | - | -** | NA | - | 13 |
| 40 | 66/F | BCLU | + | + | - | -** | +** | - |  |
| 41 | 62/M | DLBCL | + | + | - | -** | +** | - |  |
| 42 | 75/M | tFL | + | + | - | -** | +** | - |  |
| 43 | 69/M | B-LBL | + | + | *BCL6*-r | +** | -** | - |  |
| 44 | 63/F | tFL | + | + | - | +partial/+low | -/- | +λ | 14 |
| 45 | 57/F | tFL | + | + | - | +/NA | -/NA | +λ*** | 15 |
| 46 | 51/F | tFL | + | + | + | +10%/NA | -/NA | +λ*** |  |
| 47 | 24/F | tFL | + | + | - | +/NA | -/NA | NA |  |
| 48 | 54/M | tFL | + | + | - | +/NA | -/NA | NA |  |
| 49 | 5/F | B-LBL | + | - | - | +/- | -/- | +κ | 16 |

**Abbreviation.** tFL: transformation from follicular lymphoma. tMCL: transformation from mantle cell lymphoma. HGBCL: high-grade B-cell lymphoma. DLBCL: diffuse large B-cell lymphoma. B-LBL: B-lymphoblastic lymphoma. BCLU: B cell lymphoma, unclassifiable. DHL: translocation of the *MYC* proto-oncogene with a concurrent *BCL2* or *BCL6* translocation as detected by fluorescence in-situ hybridization (FISH) or standard cytogenetics (karyotype) defines double-hit lymphoma. THL: the presence of concurrent *MYC*, *BCL2*, and *BCL6* translocations. IHC: immunohistochemistry. FCM: flow cytometry. +: positive. -: negative. NA: not assessed. smIg: surface membrane immunoglobulin. M: male. F: female. Lc: light chain. *Only rare cells were positive for TdT. ** It is unclear whether the results are derived from immunohistochemistry or flow cytometry. *** Expression of immunoglobulin was determined as immunohistochemical staining.
