# Supplementary material for: Case Report: Immunophenotypically diverse immature patterns, including variable TdT expression, in aggressive B-cell lymphomas and leukemia with MYC rearrangement
Source: Front Oncol. 2025 Oct 9;15:1684005. doi: 10.3389/fonc.2025.1684005 (PMC12545147; doi:10.3389/fonc.2025.1684005)
Supplement: Supplementary Table 2 — Clinical, cytogenetic, and immunophenotypic manifestation of B-ALL and BCP-ALL with concomitant MYC rearrangement. [file Table2.docx]

**Table S2. Clinical, cytogenetic, and immunophenotypic manifestation of B-ALL and BCP-ALL with concomitant *MYC* rearrangement**

| **NO.** | **Age**  **/gender** | **Histopathological subtype** | **Chromosomal rearrangement** | | | **Immunophenotype** | | | **Ref.** |
| --- | --- | --- | --- | --- | --- | --- | --- | --- | --- |
|  |  |  | ***MYC*** | ***BCL2*** | **other** | **TdT (IHC/FCM)** | **CD20 (IHC/FCM)** | **smIg** |  |
| 1 | 85/M | B-ALL | + | - | - | +~50%/- | +5%/- | + λ | Present case 4 |
| 2 | 16.1/M | BCP-ALL | + | NA | - | NA/- | NA/+ | - | 6 |
| 3 | 4.9/M | BCP-ALL | + | NA | - | NA/- | NA/+ | - |  |
| 4 | 4.2/M | BCP-ALL | + | NA | - | NA/- | NA/NA | - |  |
| 5 | 8.2/M | BCP-ALL | + | NA | - | NA/- | NA/+ | +κ,+λ* |  |
| 6 | 14.0/M | BCP-ALL | + | + | - | NA/+ | NA/- | - |  |
| 7 | 11.3/M | BCP-ALL | + | + | - | NA/+ | NA/- | - |  |
| 8 | 5.0/F | BCP-ALL | + | NA | - | NA/- | NA/- | - |  |
| 9 | 21/M | B-ALL | + | + | - | +/NA | NA/NA | +κ*** | 17 |
| 10 | 82/F | B-ALL | + | - | *BCR-ABL* | +/NA | +/+ | +κ | 18 |
| 11 | 47/F | B-ALL | + | - | - | NA/+dim | NA/- | +κ | 19 |
| 12 | 47/M | B-ALL | + | NA | NA | +60%/NA | NA/NA | +λ*** | 20 |
| 13 | 17/M | B-ALL | + | - | - | +** | NA** | +κ** | 21 |
| 14 | 2.5/F | B-ALL | + | - | *ETV6-RUNX1* | NA/+54% | NA/+17% | +Igμ | 22 |
| 15 | 3/M | B-ALL | + | - | *MLL*-r | NA/+ | NA/- | +λ | 23 |
| 16 | 70/M | B-ALL | + | - | - | +diffuse/+ | -/(dim/absent) | +λ | 24 |
| 17 | 56/M | B-ALL | + | - | - | +10%/+<5% | -/+15% | - |  |
| 18 | 44/M | B-ALL | + | - | - | +diffuse/NA | +/+ | - |  |
| 19 | 14/M | BCP-ALL | + | - | - | NA/+ | NA/- | - | 25 |
| 20 | 13/F | BCP-ALL | + | - | - | NA/+ | NA/ (-/+) | - |  |
| 21 | 14/F | BCP-ALL | + | - | - | NA/ (-/+) | NA/ (-/+) | - |  |
| 22 | 6/M | BCP-ALL | + | - | - | NA/+ | NA/+ | - |  |
| 23 | 12/F | BCP-ALL | + | NA | - | -** | +** | - | 26 |
| 24 | 4/M | BCP-ALL | + | NA | - | NA/+ | NA/- | - | 27 |
| 25 | 10/F | BCP-ALL | + | NA | - | -** | +** | - | 28 |
| 26 | 8/F | BCP-ALL | + | NA | - | NA/+61% | NA/- | - | 29 |
| 27 | 2.5/M | BCP-ALL | + | - | - | NA/- | NA/- | - | 30 |
| 28 | 9.9/F | BCP-ALL | + | NA | NA | NA/- | NA/+ | - |  |
| 29 | 7.5/F | BCP-ALL | + | - | NA | NA/+ | NA/- | - |  |
| 30 | 5.0/F | BCP-ALL | + | - | NA | (-/+) /NA | +/NA | - |  |
| 31 | 13.4/M | BCP-ALL | + | - | - | NA/+ | NA/- | - |  |
| 32 | 0.8/F | BCP-ALL | + | - | NA | NA/- | NA/- | - |  |
| 33 | 5.5/M | BCP-ALL | + | - | - | NA/+ | NA/+ | - |  |
| 34 | 8.5/F | BCP-ALL | + | NA | NA | +/NA | -/+/NA | - |  |
| 35 | 15.4/F | BCP-ALL | + | - | - | NA/- | NA/+ | - |  |
| 36 | 11.6/F | BCP-ALL | + | NA | NA | NA/+ | NA/- | - |  |
| 37 | 14.3/F | BCP-ALL | + | - | - | NA/+ | NA/- | - |  |
| 38 | 11.6/F | BCP-ALL | + | - | - | NA/+ | NA/NA | - |  |
| 39 | 4.7/M | BCP-ALL | + | NA | NA | NA/- | NA/+ | - |  |
| 40 | 15/M | BCP-ALL | + | + | - | +/NA | NA/NA | - | 31 |
| 41 | 10/F | BCP-ALL | + | + | - | NA/+ | NA/- | - | 32 |
| 42 | 15/F | BCP-ALL | + | + | - | +/+ | +/+ | - | 33 |
| 43 | 18/M | BCP-ALL | + | + | - | NA/+ | NA/+dim | - |  |
| 44 | 24/F | BCP-ALL | + | + | - | NA/+ | NA/- | - |  |

**Abbreviation.** B-ALL: B-lymphoblastic leukemia. BCP-ALL: B-cell precursor acute lymphoblastic leukemia, that express B-cell lineage marker, CD19, 20, 22 ,79a, and terminal deoxynucleotidyl transferase and absent surface immunoglobulin. *False positive: these results were considered false positives because 𝜅 and 𝜆 were positive simultaneously. ** It is unclear whether the results are derived from immunohistochemistry or flow cytometry. *** Expression of immunoglobulin was determined as immunohistochemical staining.
